# Cellular and ultrastructural characterization of the grey-morph phenotype in southern right whales (*Eubalaena australis*)

**DOI:** 10.1371/journal.pone.0171449

**Published:** 2017-02-07

**Authors:** Guy D. Eroh, Fred C. Clayton, Scott R. Florell, Pamela B. Cassidy, Andrea Chirife, Carina F. Marón, Luciano O. Valenzuela, Michael S. Campbell, Jon Seger, Victoria J. Rowntree, Sancy A. Leachman

**Affiliations:** 1 Huntsman Cancer Institute, Salt Lake City, Utah, United States of America; 2 University of Georgia, Athens, Georgia, United States of America; 3 Department of Pathology, University of Utah, Salt Lake City, Utah, United States of America; 4 Department of Dermatology, University of Utah, Salt Lake City, Utah, United States of America; 5 Department of Dermatology, Oregon Health & Science University, Portland, Oregon, United States of America; 6 Programa de Monitoreo Sanitario Ballena Franca Austral, Puerto Madryn, Chubut, Argentina; 7 Department of Biology, University of Utah, Salt Lake City, Utah, United States of America; 8 Instituto de Conservación de Ballenas, Buenos Aires, Argentina; 9 Consejo Nacional de Investigaciones Científicas y Técnicas, Facultad de Ciencias Sociales, Universidad Nacional del Centro de la Provincia de Buenos Aires, Buenos Aires, Argentina; 10 Department of Pediatrics, University of Utah, Salt Lake City, Utah, United States of America; 11 Cold Spring Harbor Laboratory, Cold Spring Harbor, New York, United States of America; 12 Ocean Alliance/Whale Conservation Institute, Gloucester, Massachusetts, United States of America; University of Alabama at Birmingham, UNITED STATES

## Abstract

Southern right whales (SRWs, *Eubalena australis*) are polymorphic for an X-linked pigmentation pattern known as grey morphism. Most SRWs have completely black skin with white patches on their bellies and occasionally on their backs; these patches remain white as the whale ages. Grey morphs (previously referred to as partial albinos) appear mostly white at birth, with a splattering of rounded black marks; but as the whales age, the white skin gradually changes to a brownish grey color. The cellular and developmental bases of grey morphism are not understood. Here we describe cellular and ultrastructural features of grey-morph skin in relation to that of normal, wild-type skin. Melanocytes were identified histologically and counted, and melanosomes were measured using transmission electron microscopy. Grey-morph skin had fewer melanocytes when compared to wild-type skin, suggesting reduced melanocyte survival, migration, or proliferation in these whales. Grey-morph melanocytes had smaller melanosomes relative to wild-type skin, normal transport of melanosomes to surrounding keratinocytes, and normal localization of melanin granules above the keratinocyte nuclei. These findings indicate that SRW grey-morph pigmentation patterns are caused by reduced numbers of melanocytes in the skin, as well as by reduced amounts of melanin production and/or reduced sizes of mature melanosomes. Grey morphism is distinct from piebaldism and albinism found in other species, which are genetic pigmentation conditions resulting from the local absence of melanocytes, or the inability to synthesize melanin, respectively.

## Introduction

**Fig 1:**
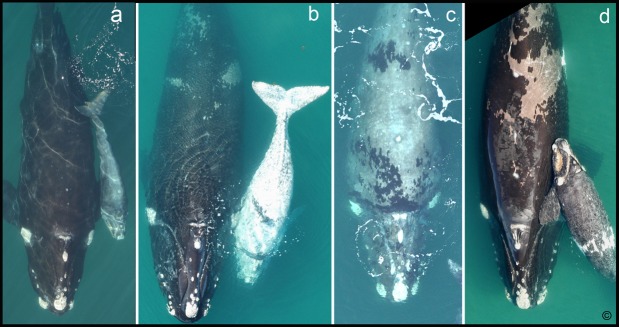
Wildtype, grey-morph and partial grey-morph phenotypes of southern right whales. *Wild-type* adults (panel a) have black skin and often have white skin patches on their bellies. *Grey morphs* (calf in panel b) are primarily white at birth with splatterings of rounded black spots that extend dorso-laterally around their bodies (calf in panel b). Their white skin becomes light grey or brown with age (panel c). *Partial grey morphs* are primarily black with splatterings of white skin at birth (calf in panel d) which also darkens with age (adults in panels b and d).. (Photos: J. Atkinson, Ocean Alliance).

Southern right whales (SRWs, *Eubalaena australis*) [1] can be individually identified from complex patterns formed by raised patches of calloused skin (callosities) on their heads, and sometimes from patterns of white skin pigmentation [2–4] (Fig 1). Callosities are grey in color but appear white from afar because they are colonized by dense populations of whale lice which are nearly white [3, 4]. These patterns are unique to individuals and are not associated with any pigmentation phenotypes. Most SRWs are primarily black (wild-type) with white belly patches that do not change through their lifetimes (Fig 1a). However, alternative pigmentation phenotypes do occur (Fig 1b–1d). Grey morphs (previously referred as “partial albinos”) are a distinctive phenotype unique to SRWs in which newborns appear mostly white with splatterings of black ovals that are often clustered in dorso-lateral bands that extend around the side of the animal (Fig 1b). Partial grey morph phenotypes also exist in which the calves have lateral bands of white splatterings on their backs (Fig 1d) [5, 6]. The white areas of the grey morph and partial grey morph skin gradually darken with age to a grey or light brown color (Fig 1b mother, Fig 1c and 1d mother). Mothers of grey morphs exhibit either grey morphism (Fig 1c) or partial grey morphism (Fig 1b and 1d).

The skin texture, thickness and eye color of grey-morph whales have not been reported to differ from those of wild-type SRWs. In other mammals, pigmentation phenotypes can impact cells of neural crest derivation and can affect internal organs, hearing, and other neurological tissues, but grey-morph and partial grey-morph SRWs appear to have a normal lifespans, normal reproductive success, and normal behavior, suggesting that they do not suffer from neurological dysfunction [5–7]. Grey-morph and partial grey-morph phenotypes have been found throughout the southern hemisphere, off South Africa, Australia and Argentina, but have not been recorded in the other two right-whale species that live in the North Pacific and North Atlantic oceans (*E*. *japonica*, *E*. *glacialis*) [8].

Schaeff et al. (1999) examined the inheritance pattern of the grey-morph and partial grey-morph phenotypes by estimating the proportions of different pigmentation patterns in 1,739 cow-calf pairs and the sexes of a subset of those animals. All 42 grey-morph calves were offspring of grey-morph or partial grey-morph cows. Among 97 SRWs of known sex, all 32 partial grey-morph individuals were females and all 14 grey-morph individuals were males. Based on these data, Schaeff et al. (1999) proposed an X-linked pattern of inheritance for grey morphism (“G” representing the wild-type [black] allele and “g” representing the grey-morph allele). Under this model, the X^G^X^G^ and X^G^Y genotypes would produce black females and males, respectively; X^G^X^g^ would produce partial grey-morph females; and X^g^X^g^ and X^g^Y would produce grey-morph females and males, respectively. This model explained the pattern of inheritance for the majority of cow-calf pairs, with the exception of two black calves that were associated with grey morph-appearing cows that were identified after the original analysis was complete (noted in proof). The authors suggest that this observation could result from inaccurate phenotyping of those pairs, partial penetrance, X-inactivation, or from the existence of an X^D^ chromosome with a deleted G locus. Apart from these two anomalous cases, the cow-calf associations imply that the grey-morph and partial grey-morph phenotypes are associated with the same X-linked gene.

Forward genetic screening has often been used to investigate phenotypic variation, gene function and dysfunction [9–10] in pigmentary disorders where the phenotypes can be readily observed [11–15]. Pigmentation gene discovery in non-human species has frequently led to a greater understanding of conserved pigment cell abnormalities in humans [16–17], including piebaldism, Waardenburg syndrome [18], and albinism [11]. Many pigmentation genes have been discovered through characterization of a pigmentary phenotype, identification of an inheritance pattern, and evaluation of microscopic and electron microscopic features of the skin and melanocytes from affected and unaffected individuals [19–22].

Additional pigmentation genes have been identified and characterized through the investigation of the various stages of melanocyte development, including derivation from the neural crest, migration and development of melanoblasts, the development of melanocyte stem cells, and proliferation of melanocytes in the skin [23–24]. Although species-specific differences in pigment cell development are likely to exist, the identification of regulators of the process may be informative with respect to understanding pigmentation patterns in the SRW. Expression of MITF (and activation of the molecular pathways downstream of this master regulator) is critical for melanocyte development [25–26]. PAX3 and SOX10 activate MITF transcription [27–29] whereas FOXD3 and SOX2 appear to repress MITF expression. Upregulation of PAX3 and SOX10 and downregulation of FOXD3 and SOX2 all appear to be critical for development of the melanoblast lineage [30–32]. FOXD3 expression is initiated by ZIC1 and PAX3 transcription factors [33] and maintained by SNAIL2 and SOX9 [31]. Upregulation of WNT3A and downregulation of BMP4 appear to be key regulators of melanoblast formation [34–35] whereas WNT3A, KIT/KIT-ligand, EDN3/EDNRB2 complexes, the EPHB2/Ephrin B ligand complex, RAC1, and P-Rex-1 are critical factors in melanocyte migration and differentiation [24]. Regulation of the process of differentiation of melanocytic cells from the neural crest is a complex, well-choreographed process and disruption of any of these critical pathways can lead to pigmentation abnormalities.

To date, three papers have been published that include investigation of melanocytes within SRW skin, but no formal direct comparison between normal and grey-morph skin has been performed. [36–38]. Histologically, SRW skin is similar to human skin, having a stratified squamous epithelium consisting of stratum corneum, stratum spinosum (spinous cell layer), and stratum basale (basal cell layer). The stratum corneum of SRWs is known to slough off or desquamate continuously in multiple layers, leaving the pitted deeper layers of the epidermis behind [36]. Martinez-Levasseur et al. [39] recently reported that fin, blue, and sperm whale species develop a presumably protective “tan” in response to UV exposure. Although no histology was described, the study reports that melanocytes seasonally increase the amount of pigmentation produced, and that blue whales may also have the capacity to increase melanocyte numbers. These features were associated with increased expression of *HSP70*, *P53*, *KIN*, and *TYR*, and were correlated with mitochondrial DNA damage, suggesting a functional UV-protective role for melanocytes in whales.

Schell et al. (2000) and Pfeiffer et al. (1996) examined SRW melanocytes using transmission electron microscopy and found that melanocytes are located primarily in the basal cell layer, have typical dendritic processes, and produce melanosomes with typical maturation and structure. Due to the challenges of sampling whales with relatively rare phenotypes, the affected skin and melanocytes of grey-morph SRWs have not been evaluated. Reeb et al. (2007) mention that grey-morph skin has fewer melanin “granules” than wild-type black skin, but this study did not further characterize, quantify or compare the melanocytes in grey-morph skin.

A primary goal of the present study was to document histologic and ultrastructural differences between grey-morph and black skinned SRWs, and to use the resulting characterization of grey-morph skin and melanocytes to restrict the range of plausible explanations for this syndrome. An improved understanding of the grey-morph phenotype in southern right whales could provide insight into the pigmentation biology of many other mammalian species, especially cetaceans.

## Materials and methods

### Phenotypic evaluation and tissue collection

SRW skin samples were collected by the Southern Right Whale Health Monitoring Program during necropsies of whales that died and stranded at Peninsula Valdes, Argentina in 2007–2008 during a period of high calf mortality [40]. SRWs were considered an IUCN lower risk/conservation dependent species at the time of sampling, and are now categorized as a species of “least concern”; no additional regulatory process was required for sampling in this situation. Samples were annotated and stored in 100% ethanol at -20°C [41]. Samples used in the present study were removed from ethanol and fixed in formalin (for paraffin embedding) or in glutaraldehyde paraformaldehyde (for electron microscopy). Samples came from six different individuals (four wild types and two grey morphs), as detailed in Table 1.

**Table 1:** Identities and characteristics of sampled whales.

Stranding Whale ID	Phenotype	Date of Necropsy	Age	Gender	Body Condition^1^	Location	Skin Color
04–07	Wild-type	08-11-07	Adult	Female	Fresh	Golfo Nuevo	Black
05–07	Wild-type	08-11-07	Calf	Female	Fresh	Golfo Nuevo	Black
16–08	Wild-type	08-17-08	Adult	Female	Unrecorded	Golfo Nuevo	Black
28–08	Wild-type	08-24-08	Calf	Female	Unrecorded	Golfo Nuevo	Black
20–08	Grey Morph	08-19-08	Calf	Male	Advanced Decomposition	Golfo Nuevo	White
57–08	Grey Morph	09-19-08	Calf	Male	Moderate Decomposition	Golfo San Jose	White

^1^—Carcass conditions were graded subjectively with the following definitions: **Fresh** = freshly dead / **Moderate Decomposition** = moderately decomposed but tissues largely intact / **Advanced Decomposition** = advanced decomposition with tissues little intact. Two other conditions existed, alive and mummified/skeletonized, but none of the samples we used had those body conditions [40].

### Light microscopic evaluation of SRW skin

Formalin-fixed samples were paraffin-embedded and 3–5 micron thick sections were cut and stained with hematoxylin and eosin as described by Florell et al. (2001) [42]. Specimens were visualized by light microscopy. Melanocytes were identified morphologically, as well as with Fontana-Masson silver stain, and counterstained with Nuclear Fast Red, as described by Florell et al. (2001) [42]. Immunohistochemical staining with the melanocyte specific markers S100 and HMB45 did not generate consistent results in these archived whale specimens, so they were omitted from the final analyses. These reagents are inferred to have failed owing to lack of recognition of the whale proteins by the human antibodies, solubility of the antigens during storage in ethanol, or lack of adequate antigen retrieval.

### Semi-quantitative analysis of melanocyte count

Melanocyte counts were performed on Fontana-Masson stained slides to increase specificity and sensitivity for detection of melanocytic cells. Positive cells were defined as those that showed melanin staining and at least two visible dendrites. The area of highest melanocyte density was identified by surveillance of the entire sample on each slide and cells were counted within ten high-powered (400X magnification) fields within the highest density area. In five of the high-powered fields, all of the melanocytes that met criteria were counted. In each of the remaining five high-powered fields, a 0.25 mm length of basal layer of the epidermis was identified and melanocytes along this basal cell layer were counted. These measurements were used to calculate the means and standard deviations of melanocyte numbers within each sample. Differences between the numbers of melanocytes counted in wild-type and grey-morph skin samples were assessed using T-tests.

### TEM evaluation of right whale skin

One wild-type and two grey-morph SRW skin samples were dissected near the dermo-epidermal junction to examine areas of the basal layer of the epidermis where melanocytes reside. Samples were minced and placed in glutaraldehyde-paraformaldehyde fixative, processed by the University of Utah Electron Microscopy Core Facility and examined by standard transmission electron microscopic (TEM) techniques. TEM images were photographed for future measurement of melanosome size.

Forty-five TEM images at eight magnifications (1100X, 1650X, 2100X, 2700X, 3200X, 4400X, 6500X, and 11,000X) were used to determine melanosome size and to evaluate melanosome maturation (12 images from one wild-type whale and 33 images from two grey-morph whales). In the wild-type and grey-morph specimens examined, a full range of immature, laminated/granular, and fully mature darker and uniform melanosomes were seen, but there were not enough melanosomes observed in all stages to permit a formal statistical analysis of grey-morph melanosome maturation. Mature melanosome sizes were determined by collecting sets of 15 images representing the largest, darkest melanosomes in each sample. The longest diameter of each melanosome was measured on 1100X images and converted to actual size. Average sizes and their standard errors were determined, and comparisons between the wild-type and grey-morph samples were evaluated statistically using Mann-Whitney U and Newman-Keuls tests (Statistica Software).

## Results

### Light microscopic evaluation of SRW skin

**Fig 2:**
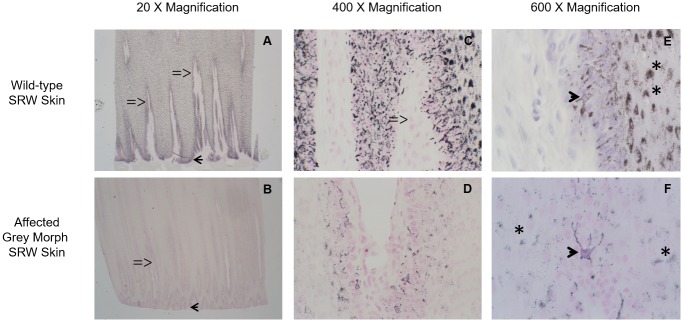
Light microscopy reveals reduced pigmentation and a decreased number of melanocytes in the affected skin of grey-morph SRWs. A comparison of Fontana Masson melanin staining is shown in wild type (Panels A, C, and E) versus grey-morph SRWs (Panels B, D, and F) at low-power (20X, Panels A and B), high-power (400X, Panels C and D), and extra high-power (600X, Panels E and F) magnification. Low-power magnification shows less melanin staining, particularly in the melanocytes distributed along the basal layer of the epidermal rete ridges (arrowheads, Panels A and B). Rete ridges and dermal papillae appear similar in grey-morph and wild-type whales. Dermal papillae are marked with “= >” in panels A, B and C. High-power magnification shows reduced melanin content and fewer positively-stained cells in the grey-morph skin (Panels C and D). Extra high-power magnification shows typical melanocyte dendrite morphology (arrowhead, Panels E and F) and normal melanosome transfer and capping of keratinocyte nuclei (asterisks, Panels E and F).

As expected, wild-type skin samples were dramatically darker than grey-morph samples (black versus off-white to grey). Light microscopy of SRW melanocytes showed very similar anatomical structure of the skin with no apparent differences in rete ridge, dermal papilla, or vascular structures. Fontana Masson melanin staining was substantially darker in wild-type animals, though staining intensity was not quantified (Fig 2). The cellular morphology of melanocytes from both grey-morph and wild-type animals was similar to benign human melanocytes. Specifically, all SRW melanocytes were located along the basal layer of the epidermis, had dendrites that made contact with surrounding keratinocytes, and had keratinocytes that demonstrated classic “capping” of nuclei in which melanosomes cluster above the nucleus (presumably for protection against ultraviolet radiation, Fig 2).

### Melanocyte counts are reduced in grey-morph relative to wild-type SRW skin

**Fig 3:**
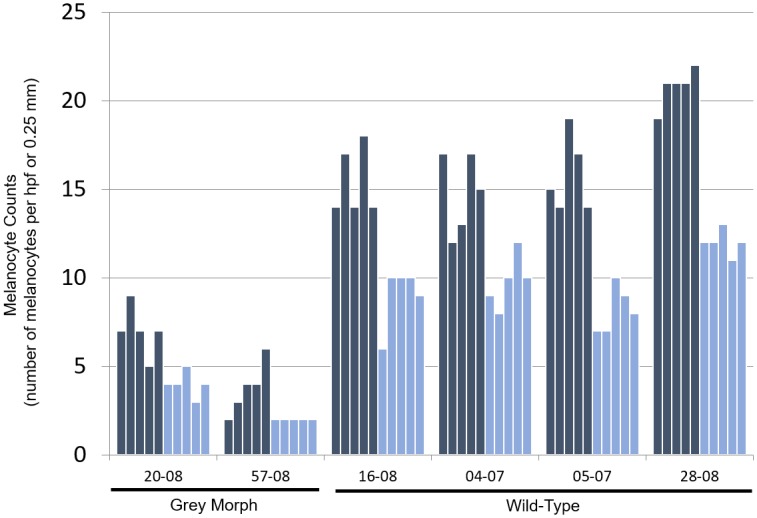
Melanocyte counts are reduced in grey-morph relative to wild-type SRW skin. Bars represent the absolute number of melanocytes counted in grey-morph and wild-type whales. Darkly shaded bars represent the number of melanocytes in each of 5 high-powered fields and lightly shaded blue bars represent the number of melanocytes along each 0.25 mm of the basement membrane measured. Data were compared for both measurement methods using T-tests. Both tests yielded p-values smaller than 1x10^-5^.

Light microscopic fields with areas of highest melanocyte density at 400X magnification were examined and the mean numbers of melanocytes in grey-morph skin were compared to wild-type SRW skin (Fig 3). The number of melanocytes in grey-morph whales ranged from 2–9 melanocytes/hpf and 2–5 melanocytes/0.25 mm basement membrane. In contrast, wild-type SRWs ranged from 12–22 melanocytes/hpf and 6–13 melanocytes/0.25 mm basement membrane. Thus the largest number of melanocytes counted in any field for grey-morph whales was less than the smallest number counted in any field for the wild-type whales, and these differences were highly significant with p-values from the T-tests for each count method being less than 1x10^-5^.

### Grey-morph melanosomes are smaller than wild-type melanosomes

**Fig 4:**
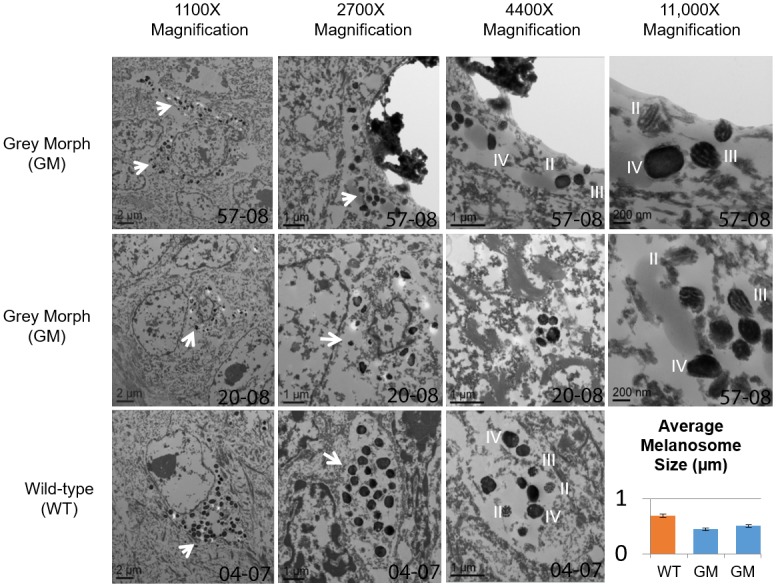
Transmission electron microscopy (TEM) reveals smaller melanosomes with normal maturation in grey-morph (GM) skin. TEM from wild-type (WT, 04–07) and GM skin (57–08 and 20–08) are shown at 1100X, 2700X, 4400X and 11,000X magnification. All skin samples showed apparently normal melanosome maturation (stage II, III, and IV melanosomes indicated by Roman numerals). The melanosomes in WT skin are larger than those found in GM skin (arrows). The mean size of fifteen Stage IV melanosomes from each animal is shown (error bars represent standard error from the mean). Mann-Whitney U and Newman-Keuls testing revealed statistically significant (p < 0.05) differences between WT and both GM melanosomes, but not between the two GM animals.

Identifying melanocytes in transmission electron microscopy (TEM) images was more difficult in the grey-morph samples, providing additional evidence that fewer melanocytes are present in grey-morph skin. Although it was not possible to determine whether grey-morph melanosomes matured in an identical way to wild-type SRWs due to a small sample size of early-stage melanomsomes, both of the grey-morph samples (20–08 and 57–08) examined by TEM contained fully mature melanosomes, suggesting that the maturation process was functional, though perhaps not completely normal since the melanosomes were smaller. Despite the smaller average size of the grey-morph melanosomes, the shape and character of melanosomes from both grey-morph and wild-type whales were similar. All samples had melanosomes that were somewhat variable in size and shape, but were predominantly spherical with both vesicular and lamellar patterns, suggesting that the agouti status and maturation process was not dramatically different in the grey-morph animals [43]. TEM images of grey-morph melanosomes showed them to be smaller qualitatively and quantitatively (Mann-Whitney U and Newman-Keuls tests (Statistica Software, Fig 4)). The wild type whale 04–07 had an average melanosome size of 0.685 μm (standard deviation 0.11 μm). Grey-morph 20–08 had an average melanosome size of 0.444 μm (standard deviation 0.09 μm). The other grey morph, 57–08, had an average melanosome size of 0.504 μm with a standard deviation of 0.1 μm. Mann-Whitney U comparisons between wild-type 04–07 and grey morphs 20–08 and 57–08 gave p = 1.4x10^-5^ and p = 2.8x10^-4^, and Newman-Keuls tests p = 1.0x10^-6^ and p = 1.5x10^-5^, respectively. Samples from the two grey-morph whales did not differ significantly (p = 0.14 and p = 0.11 for Mann-Whitney and Newman-Keuls tests, respectively) (Fig 4).

## Discussion

The goal of this study was to characterize differences between grey-morph and wild-type SRW skin, to gain insight into the biology of the condition and to develop hypotheses regarding potentially causative genes. The lighter skin of the grey morph is associated with fewer and smaller melanosomes. We were unable to perform a direct, quantitative analysis of melanin in the skin. It is possible that the absolute amount of melanin and/or number of melanosomes produced by melanocytes may also be smaller, but this could not be quantified in this small data set. Grey-morph skin had healthy, normal-appearing melanocytes with typical dendritic morphology that was appropriately localized in the basal layer of the epidermis. The melanocytes also had melanosomes that appear to have matured normally by progressing through typical stages of development.

Decreased melanosome size in grey-morph skin could be caused by a reduction in the melanin-producing capacity of melanosomes. Alternatively, the smaller sizes of grey-morph melanosomes could be the primary defect, resulting in an inability to store normal amounts of melanin. Lighter colored or earlier staged melanosomes were not abundant suggesting a primary defect in melanin synthesis. The melanin produced by grey-morph melanocytes appears to be transferred and localized appropriately in keratinocyte nuclear caps, suggesting that the melanocytes retain normal melanin transport and transfer activities. Several human disorders of pigmentation are associated with reduced ability to completely assemble functional melanosomes, including Hermansky Pudlak syndrome, Chediak-Higashi syndrome, and Griscelli syndrome. All of these conditions are associated with systemic findings including bleeding disorders, predisposition toward infections, and neurological defects secondary to abnormal biogenesis and trafficking of lysosomes (the pigmentation effect is caused by abnormal functioning of the melanosome, a modified lysosome). It is unlikely that grey-morph individuals have one of these defects because other systemic abnormalities have not been noted in such whales when observed alive or during necropsies. Grey-morph males, although not often observed in adulthood, are assumed to survive as well as wild-type individuals because of the consistent frequency of the phenotype within populations [6].

There are several possible causes for the reduced number of melanocytes seen in the affected skin of grey-morph individuals. The decreased number of melanocytes could be due to an inability to migrate effectively or to differentiate successfully during embryonic development of the neural crest. A more complete picture of the process of melanocyte development is beginning to emerge through the application of mathematical models to the process of morphogenesis [44–45] and through the identification of genes that may act as morphogens in melanocyte development [23–24]. Formal testing of these models, using relevant morphogens, may allow further understanding of the normal and abnormal pigmentation patterns observed in nature. For example, there is precedent for this pattern in piebaldism caused by inactivating mutations in *KIT* in mice and humans [46]. Piebaldism, also originally termed “partial albinism,” is characterized by well-demarcated white patches of skin localized most distally along the melanocyte migration pathway, primarily in the forelock area of the forehead and extensor knee and elbow areas [47]. It is not associated with other neurologic or systemic symptoms and the failure to migrate is believed to be caused primarily by a failure of these melanocytes to survive long enough to completely migrate, or failure to proliferate enough to reach their intended destination [48].

Grey morphism is similar to piebaldism in that there are no other associated systemic findings or neurologic issues that have been identified. The band of black on the dorsal aspect of grey morphs may represent the only successful area of melanocyte migration, but the scattered black spots elsewhere suggest that this may not be a similar migration defect. However, piebaldism also frequently has areas of hyperpigmentation within the depigmented extensor areas, which may represent partial success in migration with compensatory hyperpigmentation, and this could correspond to the scattered black spots seen in the grey morphs. The white areas of piebaldism are typically devoid of melanocytes, whereas grey-morph skin has them.

*KIT* pathways have profound effects on melanocyte function. Inactivating mutations of *KIT* lead to piebaldism, [46] whereas activating mutations in *KIT* lead to melanoma [49] and mutations in the *KIT* ligand (*KITLG*) cause the Steel phenotype in mice and progressive familial hyperpigmentation in humans [50]. Mutation of the zinc finger transcription factor *SNAI2* has also led to a piebald phenotype, but this gene is located on human chromosome 8q11.21 and the mechanism by which the mutation leads to piebaldism is poorly understood [51]. The X chromosome is relatively well conserved across mammalian species, implying that the causative gene in right whales is likely to be X-linked in other species as well [6]. The *KIT* gene is located on human chromosome 4q12, not the X-chromosome, but two microRNAs (miR221 and miR222) that inhibit *cKIT* have been identified on the human X-chromosome. If these miRNAs are conserved in whales and X-linked, they would be obvious candidate genes [52].

Other loss-of-pigment phenotypes resemble the grey-morph pattern, but to a lesser extent. For example, four subtypes of Waardenburg syndrome have been described and are known to be caused by mutations in *PAX3* (chromosome 2q35), [53–56], *MITF* (chromosome 3p14-p13) [57–58], *SOX10* (chromosome 22q13.1) [59–61], *SNAI2* (chromosome 8q11.21) [62], *EDNRB* (13q22) [63], and *EDN3* (chromosome 20q13.32) [64–65]. None of these genes are located on the X-chromosome and Waardenburg syndrome is almost always associated with systemic findings that involve dysmorphic features, deafness and other neurologic abnormalities, or megacolon, none of which are seen in grey morphs. *SOX10*, *MITF* and *EDNRB* have also been associated with additional pigmentary disorders including peripheral demyelinating neuropathy (*SOX10*), Yeminite deaf-blind hypopigmentation syndrome (*SOX10*), Tietz syndrome (*MITF*), and albinism, black lock, cell migration disorder of neurocytes and deafness syndrome (*EDNRB*); but these are clearly more debilitating and involve multiple organ systems in a pattern not present in grey-morph SRWs [66].

Other syndromes that lead to generalized decreased or abnormal pigmentation include the oculocutaneous albinism types 1–4 syndromes (*TYR*, *OCA2*, *TYRP1*, and *SLC45A2*, respectively) [Montoliu et al. 2014], Hermansky-Pudlak syndromes (*HPS1*, *AP3B1*, *HPS3*, *HPS4*, *HPS5*, *HPS6*, *DTNBP1*, *BLOCK1S3*, and *PLDN*) [67], Chediak-Higashi syndrome (*LYST*), Griscelli syndrome (*MYO5A*, *RAB27A*, and *MLPH*), and Menkes syndrome (*ATP7A*) [68]. However, none of these syndromes recapitulate the grey-morph phenotype. The process of melanogenesis is also regulated by exposure to ultraviolet radiation and subsequent DNA damage [69], and superimposed hormonal regulation of MC1R by MSH, ACTH and agouti signaling protein (ASIP) [43]. However, defects in these pathways would presumably lead to a generalized, rather than a fixed, patterned pigmentation defect. All of these syndromes have non-skin findings with striking features in the ocular, nervous, immune, clotting, or other organ systems. In addition, the pigmentation feature in these syndromes is diffuse and is seen throughout all the skin without spotting. With respect to the histologic features of oculocutaneous albinism, the melanocytes are present in normal numbers, but do not make pigment (or the pigment made is of a lighter color). Moreover, only one of these conditions has a gene defect located on the X-chromosome (Menkes disease, *ATP7A*). Finally, our data suggest that the causal gene is probably not essential for melanin synthesis (as most of these albinism genes are), otherwise a more dramatic difference would have been seen in the color of the melanin. A final set of candidates includes genes that regulate cell survival, senescence, migration or proliferation. X-chromosome candidates in this category include *PIR* (senescence), RAB-Family members (migration) and *FGF13* (proliferation).

Altered epigenetic interactions comprise another possible class of explanations for the grey-morph pigmentation patterns. For example, altered patterns of X-chromosome inactivation could be caused by mutation of an autosomal regulatory gene [70], or an X-linked epigenetic regulator of pigmentation that affects an autosomal locus could be mutated. As the SRW genome sequence becomes available, candidates from each of these categories should emerge, enabling relatively direct “field genetic” tests for the cause of grey morphism.

## Conclusions

Southern right-whale grey morphism is an X-linked condition in which skin that would be black changes to white or various shades of grey, depending on the sex and age of the affected individual. Although there is some hint of patterning of the residual black color in the affected whales, this pattern is not consistent and it could represent a mosaic or embryonic migration pattern. We found that the reduction of black pigment in grey-morph skin is associated with a reduction in the number of melanocytes. Examination of the melanocytes in white grey-morph skin shows that the melanosomes are smaller than the melanosomes seen in black skin from wild-type whales. This suggests that the genetic defect is one that reduces the ability of melanocytes to migrate, survive or expand in number. It is currently not known whether the white skin in wild-type SRWs has a melanocyte density similar to grey-morph white skin, or whether the black skin in grey morphs has as many melanocytes as the black skin in wild-type individuals. More than fifty candidate genes have been identified on the human and bovine X-chromosome that could be responsible for the grey-morph phenotype. Of these, miR221, miR222, PIR, and Rab-family members are leading candidates for further study. To date there has been little work on the molecular cell biology of skin and melanocytes in Cetacea. Identification of the gene(s) responsible for gray morphism in SRWs could lead to insights about pigment-cell biology that are relevant to human or other mammalian diseases.
